# Pediatric brain MRI without sedation: Optimization by simulation

**DOI:** 10.1186/s12887-026-06837-z

**Published:** 2026-04-23

**Authors:** Helene Mork-Knudsen, Carolina Pina Ayala, Lene Bjerke Laborie, Martine Valnumsen Hansen, Stein Magnus Aukland

**Affiliations:** 1 Department of Radiology, Haukeland University Hospital, Jonas Lies vei 65, Bergen, 5021 Norway; 2 Department of Clinical Medicine, University of Bergen, Jonas Lies vei 65, Bergen, 5021 Norway; 3 Mohn Medical Imaging and Visualization Centre, Department of Radiology, Haukeland University Hospital, Jonas Lies vei 65, Bergen, 5021 Norway

**Keywords:** Magnetic resonance imaging, Simulation training, Mock scanner, Patient education, Children, Anesthesia

## Abstract

**Background:**

MRI in young children often requires general anesthesia (GA) due to distress and movement. While GA in general is considered safe, its use introduces risks and logistical challenges. Recent research indicates that simulation-based preparation using a mock MRI scanner may reduce the need for GA.

**Objective:**

To evaluate the role of a mock MRI scanner in preparing children aged 3–6 years for awake brain MRI scans.

**Materials and methods:**

Forty children aged 3–6 years scheduled for brain MRI under GA were recruited. Each child underwent a simulation session in a mock MRI scanner. Children who completed the simulation proceeded to awake MRI. Data on awake MRI performance, image quality, and scan logistics were collected.

**Results:**

Thirty-six children (90%) successfully completed the simulation, all of whom (100%) completed the subsequent awake MRI without GA. All awake scans were diagnostically adequate. Simulation sessions effectively identified which children could tolerate awake MRI, enabling efficient scheduling and avoiding repeat scans. The remaining four (10%) children underwent MRI under GA.

**Conclusion:**

A mock MRI scanner integrated into a structured preparatory program appears to enable the use of awake MRI in children aged 3–6 years. This approach reduced reliance on GA, maintained image quality, and improved workflow and efficiency. These findings support the routine use of simulation-based preparation in pediatric radiology.

**Graphical Abstract:**

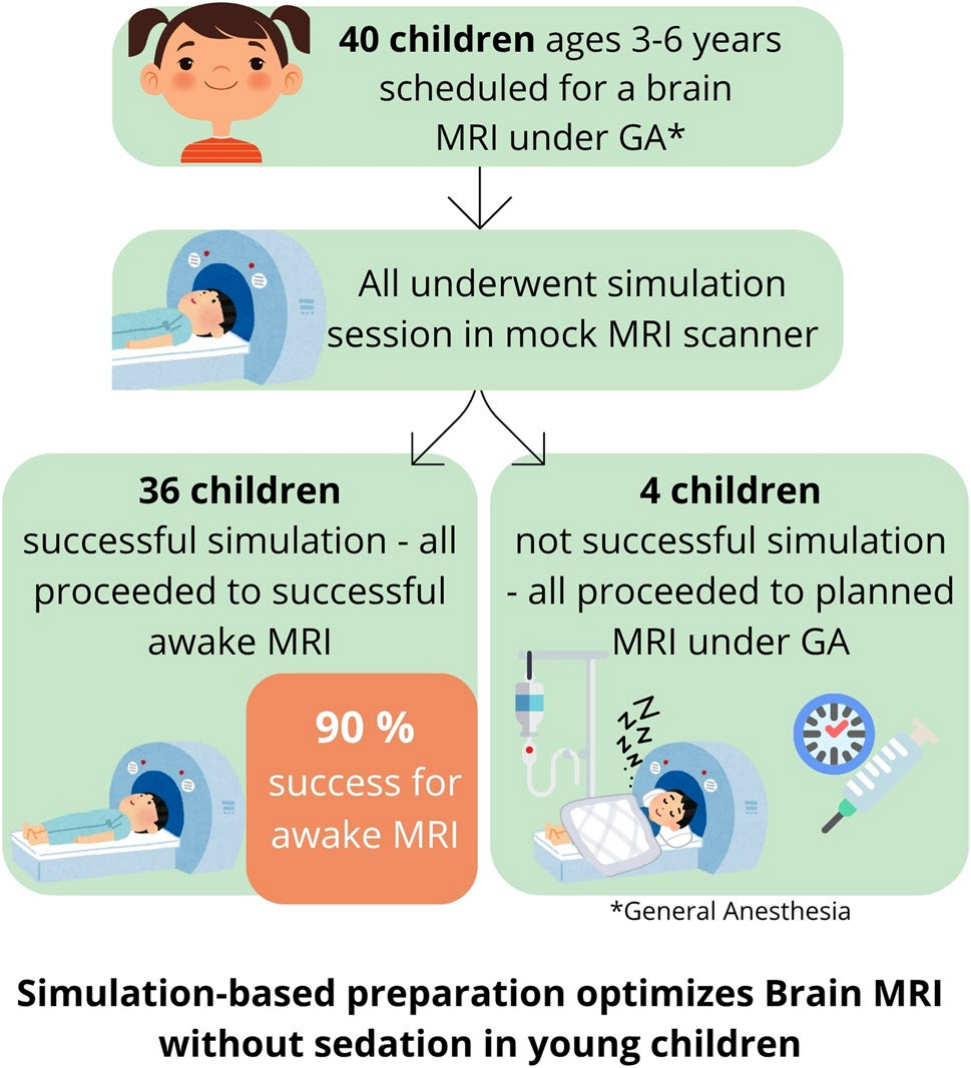

**Supplementary Information:**

The online version contains supplementary material available at 10.1186/s12887-026-06837-z.

## Introduction

Magnetic Resonance Imaging (MRI) is being increasingly used in the diagnostic work-up in children, where one of its major advantages is that the use of ionizing radiation is avoided. However, MRI might appear frightening for young children due to the in-scanner environment with loud noises, narrow space and separation from the caregivers. In addition, it may be challenging to lay still during the long scan period [1]. Consequently, sedation with general anesthesia (GA) is often routinely used in children younger than 6 to 8 years to enable a successful MRI examination [2]. Sedation involves a reduced level of awareness that can vary from light to deep, while GA results in a complete loss of consciousness [3]. Although pediatric anesthesia in general is considered safe, the APRICOT study [4] revealed a relatively high incidence of severe critical events in children undergoing GA with an overall rate of 5,2%. Hence, sedation by GA in children should be avoided whenever possible.

Our pediatric department has installed Norway´s first clinical MR Simulator - a real size mock scanner. A mock scanner is a replica unit of an MRI scanner, without magnets, in a real MRI environment. Implementing a mock scanner in the preparation program for MRI scans may improve psychological distress, especially in children [5]. By implementing a mock MRI scanner, this study seeks to prospectively evaluate whether simulation-based preparation can optimize awake MRI procedures in pediatric patients - particularly those under the age of 6, who are routinely placed under GA to undergo MRI scans at our hospital.

The primary objective of the study was to evaluate the role of a clinical mock MRI scanner as a major part of a preparatory program to facilitate awake MRI examinations in young children.

## Materials and methods

Data was collected at Haukeland University Hospital from March 2024 to June 2025. Children included in this study (3–6 years) were already scheduled for a brain MRI exam (without intravenous contrast) under GA. This was a prospective, single‑center observational implementation study with consecutive inclusion of eligible patients.

### Sample size

A formal sample size calculation was not performed, as the project was designed as a feasibility‑oriented clinical implementation study. To estimate the expected recruitment, we reviewed the number of pediatric brain MRI examinations performed under GA at our institution during the five years prior to study initiation, which indicated that approximately 40 eligible patients would be available during the study period.

### Study pilot

Upon project inclusion, a small pilot of 5 children was performed, including: Three girls aged 4, 5 and 11 years old, and two boys, both aged 9 years old. The purpose of the pilot was to test all equipment, planning, booking, and simulation session content. Four children performed the simulation on a separate day ahead of the real MRI examination, while one child performed the simulation on the same day. Based on the experience from the pilot phase and recommendations from previous research [6–8], it was decided that all simulation sessions ought to be scheduled on a separate day, in close proximity to the awake MRI examination date, preferably no more than 6 days in advance.

### Inclusion pathway

Brain MRI was chosen for homogeneity and because motion control is critical; excluding contrast avoided introducing venous access-related stress and workflow differences that could confound the effect of simulation on awake scanning success. Children were excluded if there were any contraindications for MRI i.e. MRI safety concerns such as medical implants or conditions incompatible with MRI. Additional exclusion criteria included inability to communicate sufficiently in Norwegian or English, severe developmental disorders defined as conditions that would prevent meaningful instruction or cooperation during simulation‑based preparation (e.g., inability to follow simple calm‑still instructions), inability to remain still during scanning due to medical conditions, or urgent medical needs requiring immediate MRI without time for simulation. Mild-moderate developmental delays, autism spectrum conditions, and attention difficulties were not considered exclusionary, as these children were expected to benefit from preparation and cooperative scanning. Mild communication difficulties were also not considered exclusionary when caregivers reported that communication was sufficient for the child to participate meaningfully in the session. Finally, children were excluded if they had prior awake MRI experience.

All incoming referrals for brain MRI without intravenous contrast in children aged 3–6 years were reviewed prospectively as part of the routine departmental workflow. In this way, the clinical referral pathway served as the source for identifying potential project participants, rather than external recruitment or advertising. Following referral review, a dedicated study-radiographer contacted caregivers by phone to verify eligibility using a standardized screening checklist, ensuring consistent application of inclusion and exclusion criteria. This checklist is provided in Online Resource 1.

A total of 42 consecutive patients were eligible for inclusion. However, caregivers of two possible project participants rejected inclusion in the study; one preferred to keep planned MRI under GA, without simulation in advance, and the other did not believe the child would be able to manage MRI without GA.

**Fig. 1:**
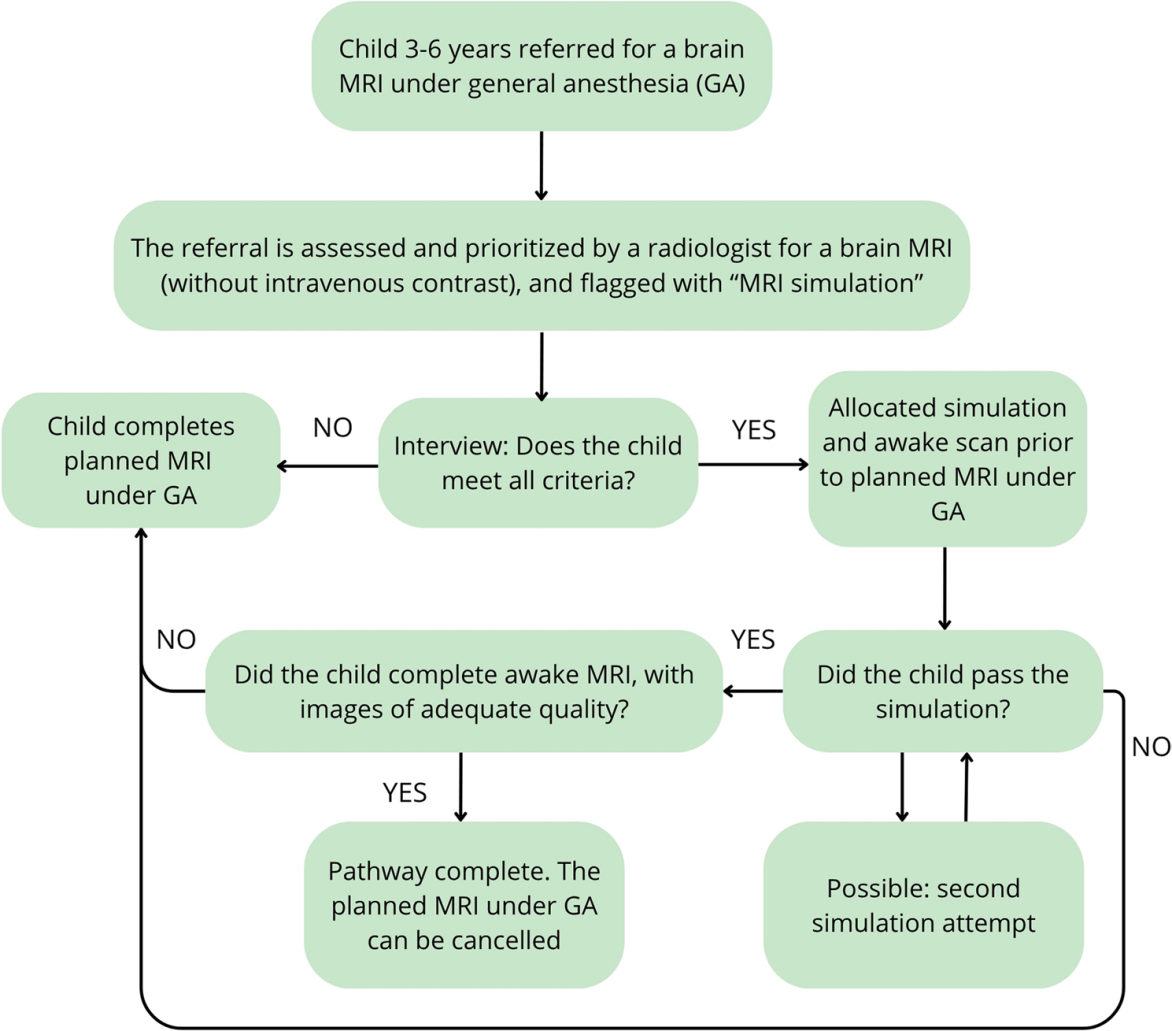
Participant inclusion and pathway for MRI with or without general anesthesia (GA)

The participants were scheduled for simulation in the mock scanner, followed by an awake MRI appointment. Both appointments were scheduled ahead of the initially planned appointment for MRI under GA. This secured time to reallocate the initial GA appointment to another child, given that the simulation and subsequent awake MRI scan for each of the study participants were successful. See Fig. 1.

### Simulation team

The simulation team consisted of three MRI study-radiographers with 4–10 years of specialized experience in imaging of young children. Whenever possible, the same radiographer who conducted the simulation session was also in charge of the child’s awake MRI scan, ensuring continuity and familiarity.

### Preparation program components

As part of the preparation program available to all pediatric patients, the department provides access to a toy MRI scanner located in the waiting area. Participants were encouraged to explore and play with the toy scanner before their scheduled simulation session in the mock scanner. In addition, a brief 3-minute YouTube video demonstrating the mock scanner was developed to support caregivers and referring clinicians in explaining the study and preparing potential participants. In this study, the preparation program therefore consisted solely of the mock scanner session, complemented by optional use of the toy scanner and the informational video.

### Simulation sessions

The simulation process, based partially on methods described by Gao, Wang [8] and De Bie, Boersma [7], was structured into the following three key steps to prepare children for awake MRI scans.

### Step 1: Initial assessment and introduction

Upon entering the mock scanner room, a safety checklist was reviewed in a preparation room next door. The session then started with familiarizing the child with the scanner environment. A study-radiographer explained the importance of staying still during the MRI, as the simulation scan is like a real scan, but only lasts five minutes. Actual scan time was then emphasized in each case, as this varies with regards to the clinical question and intended image protocol. The study-radiographer used person-centered communication [9], and open-ended questions to engage the child.

### Step 2: Individually adapted simulation

The child was positioned in a head coil with small pillows for support and motion control, mirroring the clinical setup. Their caregivers were allowed to assist and to remain in the mock scanner room for reassurance. While lying in the mock scanner, the child watched a cartoon via a mirror and was exposed to authentic MRI sounds. Both the child and their caregivers were instructed to remain silent during the noise simulation. The child practiced lying still for 5 minutes. The study-radiographer visually assessed their motion during the simulation. After the simulation, they received constructive feedback including tips on how to avoid any observed movements. If the child struggled to remain still, they were motivated to try again within the same session.

### Step 3: MRI readiness evaluation

Following the simulation, the study-radiographer documented the session in the radiology imaging and workflow management system (RIS/PACS). This documentation included any observed movements, the child’s motivation, and individual agreements made that were relevant for the upcoming MRI scan (e.g., agreeing on a comfort item, preferred video/cartoon to watch on the MRI-compatible TV screen, planned “stillness intervals” with communication during these, and parental hand placement for reassurance.) If any discrepancies from the reviewed MRI safety checklist were found during the session, this was also noted. If the child was unable to complete the simulation successfully, the additional awake MRI appointment was cancelled, and the child proceeded with their originally planned MRI under GA.

### Image acquisition, adequacy and MRI suite components

Images were acquired using a 3T MRI scanner (Signa Premier, GE). This scanner system includes advanced motion correction features on 2D and 3D, as well as 3D prospective motion correction. All patients were positioned in a head coil with small pillows on each side of the headset, to reduce patient motion. Positioning in the 3T system applies the same head-coil and pillow configuration as during simulation.

Pediatric neuroradiologists determined the scan protocol and performed the clinical interpretation in accordance with standard procedures. The diagnostic imaging adequacy of the MRI examinations was assessed during the routine clinical read within a dedicated pediatric university hospital setting, based on consensus between two experienced pediatric neuroradiologists. No formal rating scale was applied; an examination was considered “diagnostically adequate” if no additional imaging or repeat session was required to answer the clinical question.

The MRI suite is well suited for children with a multimedia-system including live, projected images on the wall and the front of the scanner - as well as an MRI compatible TV screen, where the children can watch cartoons during the MRI scan.

## Results

**Fig. 2:**
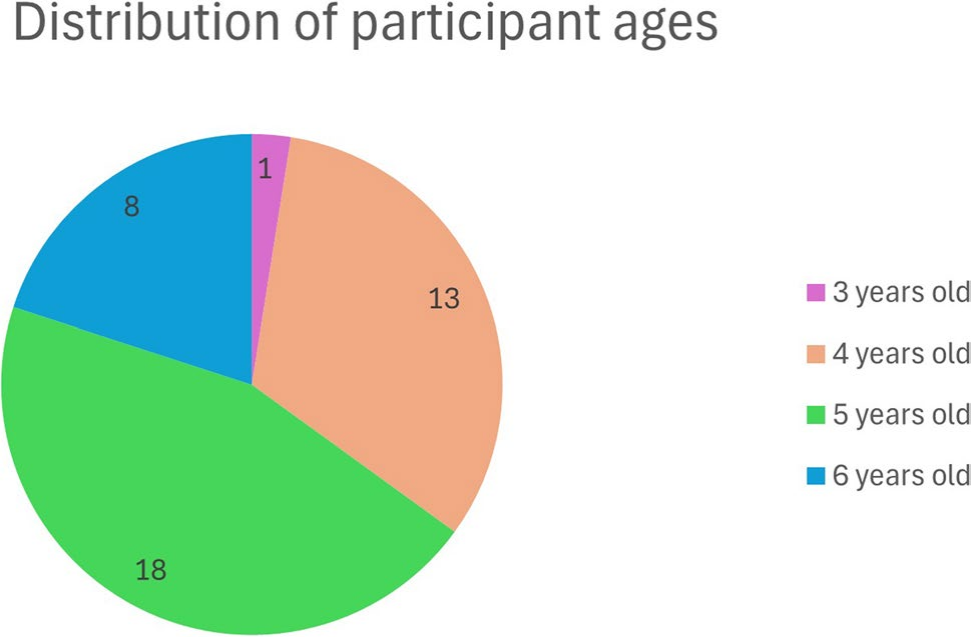
Age distribution of the 40 participants

Forty children between the ages of 3 to 6 years were included (18 girls). The mean age was 4.8 years, while the mode and median age both equaled 5 years. The age distribution is visualized in Fig. 2.

Thirty-six children (90%) successfully completed the simulation, all of whom (100%) completed the subsequent awake MRI without GA. Four children did not complete the simulation and underwent their planned MRI under GA; 2 boys and 2 girls aged 4–5 years. None of the participants were scheduled for a second simulation attempt. See Table 1 for participant study characteristics.

**Table 1:** Study characteristics of the 40 participants (18 girls, 22 boys)

Variable	Mean	Median	Range
Age	4.8 years	5 years	3–6 years
Scan time	18.3 min	18.5 min	3–34 min
Number of MRI sequences per exam	5.3 sequences	5 sequences	2–8 sequences
Time from simulation to awake MRI	1.9 days *	1 day	1–20 days
Time from simulation to planned MRI under GA	11 days	10 days	2–29 days

* One child underwent awake MRI 20 days after simulation, due to intervening holiday

**Fig. 3:**
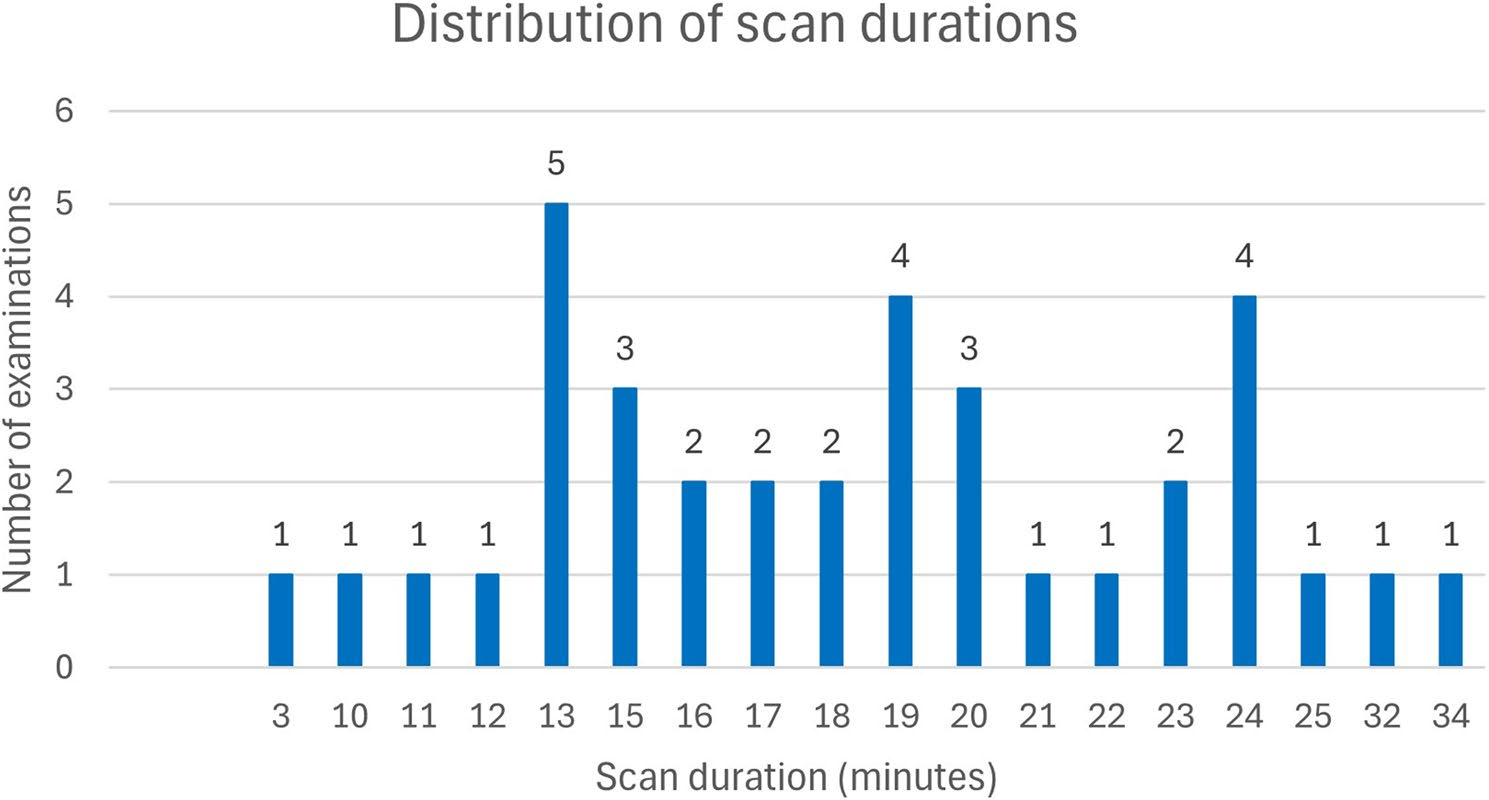
Distribution of scan durations for successful MRI scans (*n* = 36)

The mean scan time was 18.3 min, with the median being 18.5 min. Scan time distribution is visualized in Fig. 3.

All 36 MRI examinations without GA were judged to have adequate image quality. None of the 36 children required repeat scans on another day due to movement or other image quality concerns.

MRI examinations were most frequently performed for headache or headache-related concerns (*n *= 16), including suspected shunt malfunction and post-traumatic headache. A substantial number of patients underwent scheduled follow‑up for known intracranial cysts, white‑matter abnormalities, or other structural brain lesions (*n* = 8). Additional indications included delayed neurological or motor development (*n* = 4), epilepsy diagnostics or evaluation of suspected epileptic pathology (*n* = 3), and unilateral hearing loss (*n* = 3). Less common reasons for imaging were visual impairment (*n* = 1) and facial paresis (*n* = 1). 

The reasons identified for the four children who did not complete the simulation sessions were varied. Reasons included child immaturity, lack of motivation among caregivers, communication challenges due to a parent with limited Norwegian language proficiency, lack of verbal response from the child, and/or scheduling constraints on the same day because of a prior X-ray appointment.

## Discussion

A mock MRI scanner integrated into a structured preparatory program appears to enable the use of awake brain MRI in children aged 3–6 years, with 36 out of 40 children completing a simulation and a following awake MRI scan (90%). A total of 100% of the children who completed a successful simulation also completed a successful brain MRI without the need for GA.

Our findings align with previous research, demonstrating a success rate of 40–100% for awake MRI procedures in children who have participated in a variety of preparatory programs [5, 8, 10–13]. While various methods have been explored, such as mobile apps, virtual reality, and Child Life Specialists; simulation-based training using a mock scanner stands out for its ability to replicate the imaging environment.

The age of the participants was relatively well distributed, with a slight predominance of 5-year-olds. Interestingly, awake MRI performance was not limited to the older children; even among 4-year-olds, a substantial number were able to complete the awake MRI scan successfully. The study additionally included one 3-year-old, who was also scanned successfully. This could suggest that age alone should not be the sole determinant for deciding on GA, and that preparation using MRI simulations may be feasible even in younger children. The gender distribution was relatively even, with 22 boys and 18 girls, and no significant difference in awake MRI performance was observed between sexes.

Detailed neurological and behavioral profiles were not available. However, referral notes for ten children mentioned conditions such as developmental delays, autism, or attention difficulties. Despite these challenges, all children with such difficulties were still able to complete the awake MRI scan successfully. This highlights the potential of individualized simulation sessions to accommodate a wide range of cognitive and behavioral needs. Preparation with a mock scanner may be especially valuable for children with neurodevelopmental disorders, as it allows them to become familiar with the environment in a low-pressure setting, reducing sensory overload and anxiety. The use of person-centered communication, familiarization with scanner sounds, and the opportunity to choose visual entertainment likely contributed to reducing anxiety and improving compliance.

All MRI scans in this study were performed on a newly installed pediatric 3T system with advanced motion correction features and AI-based reconstruction tools. These technologies allow for shorter scan times and help maintain diagnostic image quality even with minor motion. Participants in this study had an average awake MRI scan time of 18.3 min. While some scans showed motion artifacts, all were diagnostically adequate without a need of repeat scanning. This supports the notion that minor movement during awake MRI does not necessarily compromise clinical utility, especially when balanced against the risks and logistical burdens of GA. These advanced MRI applications likely contributed to the high success and should be considered when assessing generalizability, as similar outcomes may be harder to achieve with older MRI systems.

Reducing GA use for pediatric MRI may further lead to reduced waiting times for appointments, shorter procedure times, and decreased financial costs – in addition to a reduction of the associated medical risks. In our study, the mean time from simulation to awake MRI was 1.9 days, compared with 11 days for MRI under GA. This reflects our local scheduling practices and should be interpreted as a descriptive finding rather than evidence of site-independent efficiency gains. Successful simulation may therefore potentially facilitate faster access to imaging in settings where awake MRI slots can be scheduled more flexibly than GA slots, but our data does not demonstrate a causal effect nor quantify any system-wide throughput improvement. These findings align with other relevant studies [14, 15]. This highlights the need to avoid sedation of children undergoing MR imaging whenever it is possible [4, 16]. Importantly, the simulation served as a clinical decision-making tool: children who failed the simulation went on to complete their planned MRI under GA, avoiding unnecessary stress, diagnostic delays or repeat imaging.

The total establishment cost of the simulation‑based preparation program was 622 500 NOK (≈ 65 800 USD), including installation of the mock scanner and staff time. A standard 90‑minute MRI under GA costs 16 500 NOK (≈ 1750 USD), while a 30‑minute awake MRI costs 3000 NOK (≈ 315 USD), and each child received a 60‑minute simulation session costed at 520 NOK (≈ 55 USD). These figures provide context for interpreting the economic implications of shifting selected pediatric MRI examinations from GA to awake scanning, while recognizing that a formal cost-effectiveness analysis was beyond the scope of this study. Additional potential benefits - such as increased scanner throughput, fewer cancellations, and shorter scheduling intervals - were observed but not monetized, and these findings should therefore be viewed as descriptive and context‑dependent. A formal cost‑effectiveness evaluation would be a valuable direction for future work.

Overall, the implementation of a mock MRI scanner appears to be a valuable addition to pediatric MR imaging preparation. It not only reduces the need for GA but also empowers children and their caregivers by involving them actively in the preparation process. The findings from this study support the integration of simulation-based training into routine clinical practice, particularly in departments aiming to minimize GA use in young children.

### Strengths and limitations

A key strength of the study is that simulations were carried out by a team of three trained study-radiographers, minimizing the impact of individual performance and increasing generalizability to other settings. Inclusion of children with behavioral and neurodevelopmental challenges further supports clinical relevance, although detailed neurobehavioral profiling was not available. Integration into routine practice using existing staff highlights the approach’s feasibility. No single factor was predominant in the four cases that required GA after a failed simulation attempt. Of note, two of the reasons cited for simulation failure involved limited speech or communication during the session - issues that closely overlap with the study’s exclusion criteria. In the Methods section, we define this exclusion criterion as severely limited language or communication skills that prevent meaningful instruction during simulation‑based preparation. 

Mild language barriers were not considered exclusionary based on caregiver reports during telephone screening, but in practice these reports occasionally led to borderline cases. In one instance, the caregiver attending the session differed from the caregiver screened by phone and had limited Norwegian language proficiency; in another, the child chose not to communicate despite likely having adequate abilities. These cases highlight the practical difficulty of assessing language and communication capacity in young children remotely and likely contributed to non‑completed simulations. Such inconsistencies might have been reduced through an in‑person pre‑inclusion assessment, though this was not feasible within the resource constraints of this implementation study.

This study merely provided an alternative to the scheduled pediatric MRI examinations under GA. As such, we cannot determine how many of the included children might have successfully completed an awake MRI without simulation, had they been given the opportunity. Due to high scanner demand and limited departmental resources, it was not considered feasible to routinely offer awake attempts without prior simulation-based assessment. The ideal study design would be to randomize the participants into two groups: simulation and awake MRI, or no simulation and awake MRI. The absence of a control group (e.g., children undergoing MRI without simulation) makes it difficult to isolate the specific impact of the mock scanner from other preparatory elements and/or scan components. Additionally, the absence of a standardized, multi‑reader scoring system represents a methodological limitation that should be addressed in future studies. No formal anxiety or distress scale was collected for this age group, representing a target for future studies.

Finally, the sample size was relatively small (*n* = 40), and the study was conducted within a single clinical setting, which may limit the generalizability of the results.

## Conclusion

A mock MRI scanner integrated into a structured preparatory program appears to enable the use of awake brain MRI in children aged 3–6 years. Importantly, the simulation served as a highly effective tool for assessing whether a child could successfully undergo an awake MRI. With our inclusion criteria and structured setup, we achieved a 100% success in this evaluation, underscoring the reliability of simulation as a predictive measure.

This approach reduced reliance on GA, maintained image quality, and improved workflow and efficiency. Given these outcomes, the mock MRI program has recently transitioned from a pilot initiative to an established part of our clinical practice, highlighting its feasibility and value in everyday care.

Future research should aim to validate these results in larger populations, while also considering randomized controlled designs to better assess the efficacy of simulation-based preparation with a mock MRI scanner.

## Supplementary Information

Supplementary Material 1.

## Data Availability

De-identified individual participant data will be made available. The data will be made available upon publication to researchers who provide a methodologically sound proposal for use in achieving the goals of the approved proposal. Proposals should be submitted to helene.mork-knudsen@helse-bergen.no.
