# Supplementary material for: Pediatric brain MRI without sedation: Optimization by simulation
Source: BMC Pediatr. 2026 Apr 23;26:537. doi: 10.1186/s12887-026-06837-z (PMC13244846; doi:10.1186/s12887-026-06837-z)
Supplement: Supplementary file 1 — Supplementary Material 1. [file 12887_2026_6837_MOESM1_ESM.pdf]

Supplementary materials:

Online Resource 1

Patient inclusion checklist for interview

Section	Checklist Item	Notes / Action
**Before Calling**	Check in imaging system if patient has had awake MRI before and seemingly meets inclusion criteria.<br><br>Confirm MRI is scheduled and time is available for simulation.<br>Ensure same radiographer is available for both training and awake MRI.<br><br>Check planned MRI duration. If unsure, confirm with MRI radiographer.	Note suggested time slots
**1. Inclusion Criteria – Part 1**	No contraindications for MRI (including accompanying person).<br><br>Patient speaks/understands Norwegian or English; vision and hearing adequate.<br><br>Confirm with guardians that the child has *not* done an awake MRI before.	
**2. Inclusion Criteria – Part 2**	Child can express needs and communicate sufficiently<br><br>Child is physically able to lie on their back.	
**3. About the Project & Participation**	Briefly explain the project and purpose of mock MRI.<br><br>Describe the session (approx. 1 hour; 5 mins inside mock scanner with sounds).<br><br>Explain that it's a research project and mention that all participants receive an info sheet (after simulation or awake MRI)<br>Ask permission to include in project.	

**4. Additional Information**	Ask if guardian believes this preparation will help the child.<br><br>Recommend YouTube video or online project page for more information.	Most important if other criteria are uncertain.<br><br>Link sent via SMS.
**5. Notes for Training Session**	Ask if guardian wants to share any other tips or information about the child.<br><br>Ask about child's favorite video/cartoon/TV program	Note information
**6. Schedule Appointments**	Note planned MRI duration:<br><br>Date of simulation session (mock MRI):<br><br>Date of awake MRI:<br><br>Date of planned sedated MRI appointment:	Fill in.<br><br>Fill in.<br><br>Fill in.<br><br>Fill in.
